# Multiparametric magnetic resonance imaging for the assessment of non‐alcoholic fatty liver disease severity

**DOI:** 10.1111/liv.13284

**Published:** 2017-05-30

**Authors:** Michael Pavlides, Rajarshi Banerjee, Elizabeth M. Tunnicliffe, Catherine Kelly, Jane Collier, Lai Mun Wang, Kenneth A. Fleming, Jeremy F. Cobbold, Matthew D. Robson, Stefan Neubauer, Eleanor Barnes

**Affiliations:** ^1^ Oxford Centre for Clinical Magnetic Resonance Research Radcliffe Department of Medicine University of Oxford Oxford UK; ^2^ Translational Gastroenterology Unit University of Oxford Oxford UK; ^3^ The Oxford NIHR Biomedical Research Centre Oxford UK; ^4^ Perspectum Diagnostics Oxford UK; ^5^ Department of Histopathology Oxford University Hospitals Oxford UK; ^6^ The Peter Medawar Building for Pathogen Research University of Oxford Oxford UK

**Keywords:** diagnostic accuracy, non‐alcoholic steatohepatitis, non‐invasive test, sensitivity and specificity

## Abstract

**Background & Aims:**

The diagnosis of non‐alcoholic steatohepatitis and fibrosis staging are central to non‐alcoholic fatty liver disease assessment. We evaluated multiparametric magnetic resonance in the assessment of non‐alcoholic steatohepatitis and fibrosis using histology as standard in non‐alcoholic fatty liver disease.

**Methods:**

Seventy‐one patients with suspected non‐alcoholic fatty liver disease were recruited within 1 month of liver biopsy. Magnetic resonance data were used to define the liver inflammation and fibrosis score (LIF 0‐4). Biopsies were assessed for steatosis, lobular inflammation, ballooning and fibrosis and classified as non‐alcoholic steatohepatitis or simple steatosis, and mild or significant (Activity ≥2 and/or Fibrosis ≥2 as defined by the Fatty Liver Inhibition of Progression consortium) non‐alcoholic fatty liver disease. Transient elastography was also performed.

**Results:**

Magnetic resonance success rate was 95% vs 59% for transient elastography (*P*<.0001). Fibrosis stage on biopsy correlated with liver inflammation and fibrosis (*r*_s_=.51, *P*<.0001). The area under the receiver operating curve using liver inflammation and fibrosis for the diagnosis of cirrhosis was 0.85. Liver inflammation and fibrosis score for ballooning grades 0, 1 and 2 was 1.2, 2.7 and 3.5 respectively (*P*<.05) with an area under the receiver operating characteristic curve of 0.83 for the diagnosis of ballooning. Patients with steatosis had lower liver inflammation and fibrosis (1.3) compared to patients with non‐alcoholic steatohepatitis (3.0) (*P*<.0001); area under the receiver operating characteristic curve for the diagnosis of non‐alcoholic steatohepatitis was 0.80. Liver inflammation and fibrosis scores for patients with mild and significant non‐alcoholic fatty liver disease were 1.2 and 2.9 respectively (*P*<.0001). The area under the receiver operating characteristic curve of liver inflammation and fibrosis for the diagnosis of significant non‐alcoholic fatty liver disease was 0.89.

**Conclusions:**

Multiparametric magnetic resonance is a promising technique with good diagnostic accuracy for non‐alcoholic fatty liver disease histological parameters, and can potentially identify patients with non‐alcoholic steatohepatitis and cirrhosis.

**Abbreviations**
AUROC: area under the receiver operating characteristic curve
95% CI: 95% confidence interval
CoV: coefficient of variance
cT_1_: iron‐corrected T_1_
FLIP: fatty liver inhibition of progression
LIF: liver inflammation and fibrosis
LMS: LiverMultiScan^TM^
LS: liver stiffness
MRE: magnetic resonance elastography
MRI: magnetic resonance imaging
MR: magnetic resonance
NAFLD: non‐alcoholic fatty liver disease
NASH: non‐alcoholic steatohepatitis
ROC: receiver operating curve
ROI: region of interest
SAF: steatosis, activity, fibrosis
TE: transient elastography

**Key points**

- Multiparametric magnetic resonance (MR) can be used to derive the liver inflammation and fibrosis score (LIF), a non‐invasive, quantitative score that can be used to evaluate non‐alcoholic fatty liver disease (NAFLD).
- In patients with NAFLD, LIF score had good diagnostic accuracy, both for the diagnosis of non‐alcoholic steatohepatitis and ballooning.
- The LIF score also had good diagnostic accuracy for cirrhosis.
- This methodology has the potential to be used for risk stratification in clinical practice and as a surrogate end point in clinical trials.

## 1 INTRODUCTION

Non‐alcoholic fatty liver disease (NAFLD) represents a disease spectrum ranging from accumulation of liver fat only (steatosis) to fat associated with inflammation (non‐alcoholic steatohepatitis; NASH) and fibrosis. NAFLD has now reached epidemic levels in developed countries, affecting a third of the adult population.^1^ NASH prevalence is estimated at 3%‐12%,^2^, ^3^ and is expected to become the most common indication for liver transplantation in the near future.^4^ Steatosis and NASH have been traditionally regarded as distinct disease entities with steatosis generally running a benign course and with NASH associated with disease progression.^5^, ^6^ However, some patients with simple steatosis can develop progressive disease,^7^ suggesting that NAFLD may be more complex than previously thought.

The diagnosis and classification of NAFLD into different subtypes (steatosis, NASH) and staging of fibrosis often relies on liver biopsy, and this is problematic because of the inherent drawbacks of this technique (eg sampling and observer dependent variability).^8^ Furthermore, the majority of patients with NAFLD have uncomplicated steatosis, where non‐invasive diagnosis would be preferable. There is therefore a clinical need for reliable non‐invasive biomarkers for the assessment of NAFLD.

Non‐invasive biomarkers can be broadly divided into serum based and imaging or elastography technologies. Serum biomarkers have yielded mixed results that have hindered widespread clinical application. For example, cytokeratin‐18 has demonstrated moderate overall accuracy for diagnosing NASH in a meta‐analysis (66% sensitivity, 82% specificity),^9^ but was found to have only a limited sensitivity (58%) for the diagnosis of NASH in a large clinical study.^10^

Measurement of liver stiffness (LS) using transient elastography (TE) 11 is increasingly used for the assessment of fibrosis in patients with viral hepatitis. However, it is associated with high failure rates, particularly in obese patients (BMI>30 kg/m^2^),^12^ where reliable measures could only be obtained in 65% of patients in one study.^13^ This limits the applicability of TE for the assessment of patients with NAFLD who are often obese.

Measuring liver stiffness using magnetic resonance elastography (MRE) has shown promise in the evaluation of fibrosis in patients with NAFLD,^14^ outperforming serum‐based tests and ultrasound‐based elastography techniques.^15^, ^16^ More recently, a more advanced version of this technique (3D‐MRE) has produced even better results than the commercially available 2D‐MRE.^17^ However, the accuracy of MRE for the diagnosis of NASH is limited, and this technique remains restricted to specialist centres with considerable obstacles to widespread use (eg need for additional hardware). MRI techniques that can be implemented using scanners available in routine practice offer an attractive alternative for NAFLD evaluation.

We have recently developed a multiparametric magnetic resonance (MR) technique that allows quantification of liver inflammation and fibrosis.^18^, ^19^, ^20^ This technique has shown a high diagnostic accuracy compared to histology^18^ and can also provide prognostic information^21^ in patients with mixed liver disease aetiologies.

The primary aim of this study was to evaluate the diagnostic performance of multiparametric liver MRI specifically in the assessment of patients with NAFLD using liver histology as the reference standard. We also compared this to TE in the assessment of fibrosis. The analysis was conducted using components of the steatosis, activity and fibrosis (SAF) score and the diagnostic categories of the Fatty Liver Inhibition of Progression (FLIP) consortium algorithm.^22^

## 2 PATIENTS AND METHODS

### 2.1 Study design and patient population

**Figure 1:**
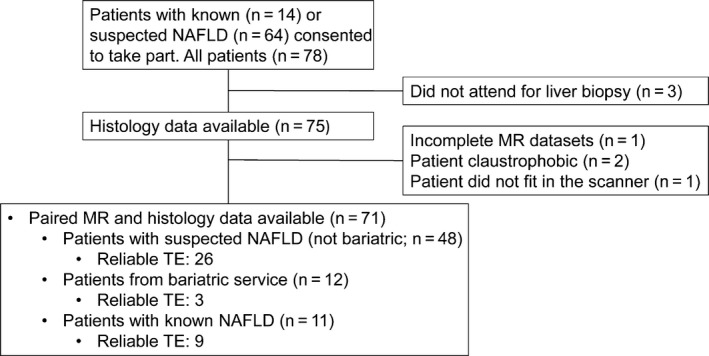
Study flow chart. NAFLD, non‐alcoholic fatty liver disease, MR, magnetic resonance, TE, transient elastography

This was a prospective pilot study conducted at a UK tertiary centre (John Radcliffe Hospital, Oxford, UK) between May 2011 and March 2015. Adult patients (≥18 years) with suspected or known NAFLD were invited to participate (see also Data S1). Patients attended for a single visit, for multiparametric MR examination, TE and blood sampling. The median (IQR) interval between the study visit and biopsy was 13^5^, ^6^, ^7^, ^8^, ^9^, ^10^, ^11^, ^12^, ^13^, ^14^, ^15^, ^16^, ^17^, ^18^, ^19^, ^20^, ^21^, ^22^, ^23^, ^24^, ^25^, ^26^, ^27^ days. All the examinations were carried out after a fasting period of at least 4 hours. Patients were recruited from general hepatology and metabolic liver disease clinics and from the bariatric surgery service. Biopsies were performed as part of normal clinical practice for the diagnosis of NASH and fibrosis staging. Figure 1 summarises the recruitment to the study.

Patients were ineligible if they had: (a) contraindications to MRI scanning, (b) consumed more alcohol than the current limit recommended by the UK Department of Health (2‐3 units [16‐24 g]/day for women and 3‐4 units [24‐32 g]/day for men), (c) had clinical or laboratory evidence of a liver diagnosis other than NAFLD, including chronic viral hepatitis (positive surface antigen for hepatitis B, or positive hepatitis C antibody), cholestatic liver disease, Wilson's disease, hereditary haemochromatosis or alpha‐1‐antitrypsin deficiency.

The study was conducted according to the ethical guidelines of the 1975 Declaration of Helsinki and was approved by a UK National Research Ethics Committee. All participants gave written informed consent for participation in the study.

### 2.2 Multiparametric MR examination

All MR scans were performed with the patient lying supine in a 3‐Tesla scanner (Siemens, Tim Trio, Germany). The individual components of the multiparametric MR protocol were T_1_ mapping and T_2_* mapping which were used to calculate the iron‐corrected T_1_ and LIF score (see also Data S1).

### 2.3 Iron‐corrected T_1_ and the liver inflammation and fibrosis score

T_1_ relaxation time increases with increases in extracellular fluid and is characteristic of fibrosis and inflammation. However, the presence of iron, which can be accurately measured from T_2_* maps, has an opposing effect on the T_1_. An algorithm has been created that allows for the bias introduced by elevated iron to be removed from the T_1_ measurements, yielding the iron‐corrected T_1_ (cT_1_).^18^, ^20^ Optimal cT_1_ cut‐off points for the differentiation of: no (Ishak fibrosis stage F0), mild (Ishak F1‐2), moderate (Ishak F3‐4) and severe (Ishak F5‐6) liver fibrosis have been derived from the association of cT_1_ with histological fibrosis in our previous study.^18^ These cut‐offs were used to develop the liver inflammation and fibrosis (LIF) score, a standardised continuous score (0‐4).

**Figure 2:**
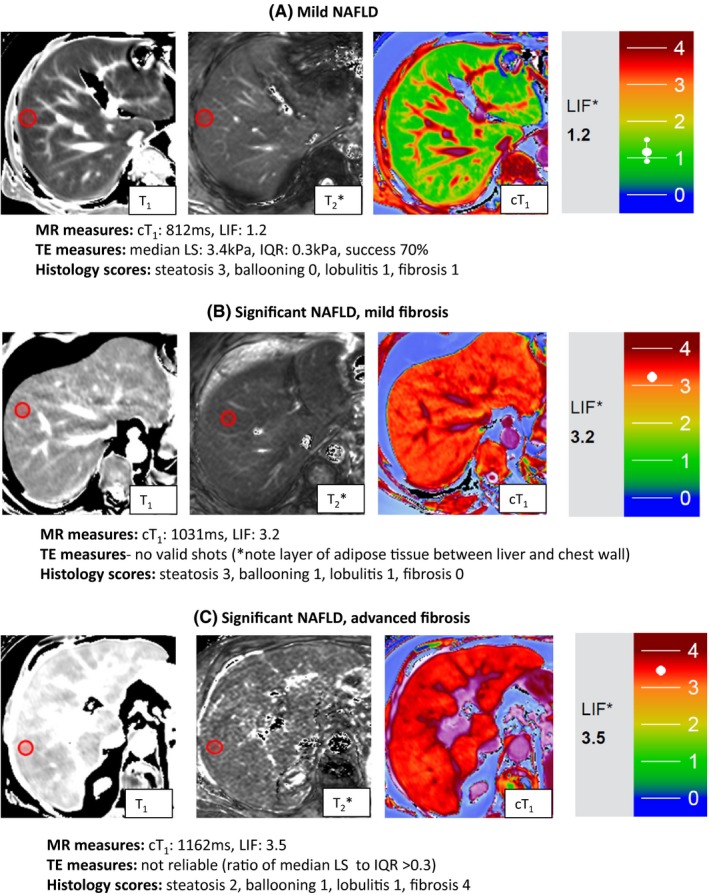
Representative magnetic resonance data. Magnetic resonance data (T_1_, T_2_*, cT_1_ maps and LIF scores) from patients classified using the Fatty Liver Inhibition of Progression (FLIP) algorithm as having: mild disease (top panel), significant disease/mild fibrosis (middle panel) and significant disease/advanced fibrosis (bottom panel). Red circles indicate typical regions of interest. Iron‐corrected T_1_ (cT_1_), liver inflammation and fibrosis (LIF) scores, and corresponding transient elastography (TE) data and histological scores are included. The predefined colour scale for generating the LIF score is based on the cT_1_ maps and is shown in each case [Colour figure can be viewed at wileyonlinelibrary.com]

LiverMultiScan™ (LMS, Perspectum Diagnostics, Oxford, UK), is a software product that can be used to measure cT_1_ and LIF scores from T_1_ and T_2_* maps. For this study, LMS was used to analyse anonymised images, by a blinded investigator (MP). Interobserver agreement was assessed in a subset of consecutive scans (see Data S1 and Figure S1). LIF scores were measured in two operator‐defined, regions of interest (ROI), one in each liver lobe, and the average value was used in the analysis. The coefficient of variance (CoV) for the measurement of cT_1_/LIF on two different occasions on the same patient (test, re‐test CoV) was previously found to be 1.8%.^18^ Figure 2 illustrates typical MR data from patients with varying disease severity.

### 2.4 Transient elastography

TE was performed using Fibroscan (Echosens, France) by operators (MP or RB) who were certified by the manufacturer to perform liver stiffness measurements. TE was performed with the patient lying supine and with the right arm fully extended. Both the medium (M) probe and extra‐large (XL) probes were used. Ten measurements per patient were needed for a successful scan and the manufacturer's recommendations were used to assess the validity of each examination (10 valid measurements; 60% success rate; interquartile range to median ratio <0.3).

### 2.5 Liver histology

Percutaneous biopsies (n=50) were performed under ultrasound guidance using 18G cutting biopsy needles and trans‐jugular (n=9) biopsies were performed under fluoroscopic guidance using 19G needles. Patients who were having bariatric surgery (n=12) had wedge liver biopsies intra‐operatively. The median (IQR) biopsy length in patients who had needle biopsies was 18 mm,^14^, ^15^, ^16^, ^17^, ^18^, ^19^, ^20^, ^21^, ^22^, ^23^, ^24^, ^25^ including a median (IQR) of 10 (7‐13) portal tracts. All biopsies were included in the final analysis.

Biopsies were evaluated by two experienced liver pathologists and discussed in a clinico‐pathological meeting before a final consensus report was issued, and this was used as the reference standard in this study. The reporting pathologists and clinicians attending the clinico‐pathological meeting were blinded to the MR data.

Biopsies were assessed for steatosis, ballooning, lobular inflammation and fibrosis. Steatosis was assessed on a 4‐tier scale (0‐3), ballooning and lobular inflammation on a 3‐tier scale (0‐2) and fibrosis on a 5‐tier scale (0‐4). The four histological components were summed into the steatosis, activity (ballooning + lobular inflammation) and fibrosis (SAF) score. Patients were categorised into steatosis and NASH (steatosis ≥1 and ballooning ≥1 and lobular inflammation ≥1) and for the overall disease severity into mild (Activity < 2 and Fibrosis < 2) and significant disease (Activity ≥2 and/or Fibrosis ≥2) according to the algorithms suggested by the Fatty Liver Inhibition of Progression (FLIP) consortium.^22^

### 2.6 Statistical analysis

All the analysis was carried out using GraphPad Prism software (version 6.05, July 7, 2014). Statistical significance was set at *P*<.05. Descriptive statistics were used to summarise baseline subject characteristics. Normality was determined using the Shapiro‐Wilks test. Associations were tested using the Spearman's correlation coefficient (*r*_s_). Differences between groups were assessed using the Mann‐Whitney test. Fisher's exact test was used to test for the differences in proportions between two groups. Differences between multiple groups were assessed using the Kruskal‐Wallis test with Dunn's correction for multiple comparisons.

Receiver operating characteristic curves (ROC) were used to determine (a) the diagnostic accuracy of multiparametric MR for the assessment of NAFLD components (ballooning, lobular inflammation, activity, fibrosis, NASH vs steatosis, mild vs significant NAFLD, and (b) the diagnostic accuracy of TE in the assessment of NAFLD fibrosis. A cut‐off to optimise sensitivity at 90% was reported. Ninety‐five percent confidence intervals (95% CI) were calculated for the parameters of diagnostic accuracy.

## 3 RESULTS

### 3.1 Baseline characteristics

A total of 78 patients consented to participate and biopsy data were available in 75. Of these, 71 (95%) had a successful MRI and were included in the final analysis (Figure 1). TE was attempted in 64 (90%) patients with valid measurements obtained in 38 (59%) patients (Figure S2). The success of TE was significantly lower than multiparametric MR (*P*<.0001). The mean (±SD) age was 53.4 years (±11.6) and patients had a median (IQR) Body Mass Index (BMI) of 32.7 kg/m^2^ (28.1‐38.1). The majority of the patients were male (n=43; 60%) and 25 (35%) had type 2 diabetes mellitus.

Categorised using the FLIP algorithm,^22^ 25 (35%) patients had steatosis and 46 (65%) had NASH. For overall disease severity, 13 (18%) patients were classed as having mild disease and 58 (82%) as having significant disease. The number of patients with fibrosis stages 0, 1, 2, 3 and 4, were 5 (7%), 20 (28%), 20 (28%), 15 (21%) and 11 (15%) respectively. The subject characteristics of the whole cohort (demographics, liver function tests, metabolic profile, histology and MR data) are presented in Table 1, and for subpopulations within the study (suspected NAFLD, known NAFLD, patients undergoing bariatric surgery) in Tables S1‐3.

**Table 1:** Baseline patient characteristics

	All patients (n=71)
Age (years; mean ± SD)	53.4 (±11.6)
Male (n, %)	43 (60)
BMI (kg/m^2^; median; IQR)	32.7 (28.1–38.1)
Type 2 Diabetes mellitus (n, %)	25 (35)
Liver function tests; median (IQR)	
Bilirubin (μmol/L)	10 (7–16)
ALT (IU/L)	54 (30–76)
ALP (IU/L)	172 (138–233)
Albumin (g/L)	45 (44–47)
GGT (IU/L)	66 (36–118)
AST (IU/L)	40 (31–56)
Haematological tests; median (IQR)	
Platelet count (x10^9^/l)	208 (166–278)
Prothrombin time (s)	13.6 (13.1–14.5)
Metabolic profile; median (IQR)	
Glucose (mmol/L)	5.1 (4.8–6.3)
Cholesterol (mmol/L)	4.7 (3.8–5.6)
HDL (mmol/L)	1.1 (1.0–1.2)
LDL (mmol/L)	2.7 (2.0–3.5)
Triglycerides (mmol/L)	1.6 (1.2–2.4)
Histology (n, %)	
Fibrosis	
0	5 (7)
1	20 (28)
2	20 (28)
3	15 (21)
4	11 (15)
Ballooning	
0	17 (24)
1	46 (65)
2	8 (11)
Lobulitis	
0	12 (17)
1	58 (82)
2	1 (1)
Steatosis	
0	4 (6)^a^
1	8 (11)
2	17 (24)
3	42 (59)
FLIP algorithm classification (n; %)	
Steatosis	25 (35)
NASH	46 (65)
Mild disease	13 (18)
Significant disease	58 (82)
Non‐invasive scores; median (IQR)	
cT_1_ (ms)	923 (832–1035)
LIF score	2.6 (1.5–3.3)
Liver stiffness (kPa; n=38)	7.5 (5.1–13.3)

FLIP, Fatty Liver Inhibition of Progression consortium; MR, magnetic resonance; cT_1_, iron‐corrected T_1_; LIF, liver inflammation and fibrosis.

a Four patients with suspected NAFLD were found to have no steatosis on liver biopsy but still included as the absence of NAFLD could not have been predicted without liver biopsy.

### 3.2 Assessment of fibrosis

#### 3.2.1 Multiparametric magnetic resonance

**Figure 3:**
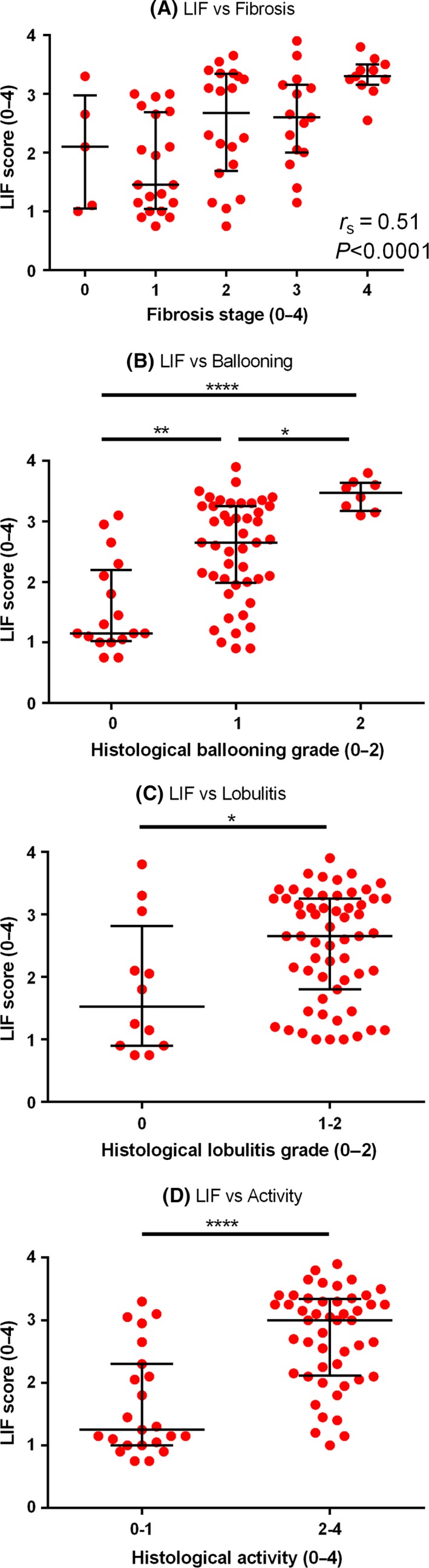
Liver inflammation and fibrosis score for the evaluation of (A) fibrosis, (B) ballooning, (C) lobular inflammation and (D) activity. (A) There was a significant association between LIF and fibrosis (*r*_s_=.51; *P*<.0001). The median LIF score could differentiate (B) ballooning grades (LIF 1.2, 2.7 and 3.5 for ballooning grades 0, 1 and 2 respectively (*P*<.05), (C) lobular inflammation grades (LIF 1.6 vs 2.7 for lobular inflammation grade 0 vs >0) and (D) overall activity (LIF 1.3 vs 3.0 for mild (0‐1) vs significant activity.^2^, ^3^, ^4^ Lines and error bars indicate the median and interquartile range on all graphs [Colour figure can be viewed at wileyonlinelibrary.com]

There was a significant association between histological fibrosis and LIF (*r*_s_=.51, *P*<.0001; Figure 3). The median LIF for patients without cirrhosis (F0‐3) was 2.2 and for patients with cirrhosis (F4) 3.3 (*P*<.0001). The area under the receiver operating characteristic curve (AUROC) for the diagnosis of cirrhosis was 0.85 (95% CI: 0.76‐0.95; *P*=.0002). A LIF cut‐off of 3.0 had a sensitivity 91% (95% CI: 59%‐100%) and specificity 73% (55%‐80%) for the diagnosis of cirrhosis; Table 2).

**Table 2:** Diagnostic parameters of liver inflammation and fibrosis score for NAFLD assessment

	AUROC (95% CI)	*P*	LIF cut‐off	Se. (%; 95% CI)	Sp. (%; 95% CI)
SAF score components^a^					
Ballooning grade 0 vs >0	0.83 (0.72‐0.93)	<.0001	1.2	91 (82‐98)	53 (28‐77)
Activity grade					
0‐1 vs >1	0.83 (0.73‐0.93)	<.0001	1.6	90 (77‐97)	61 (39‐80)
Fibrosis stage					
0‐3 vs 4	0.85 (0.76‐0.95)	.0002	3.0	91 (59‐100)	68 (55‐80)
FLIP algorithm categories^a^					
Steatosis vs NASH	0.80 (0.69‐0.92)	<.0001	1.4	91 (79‐98)	52 (31‐72)
Mild vs significant	0.89 (0.80‐0.98)	<.0001	1.4	90 (79‐96)	77 (46‐95)

AUROC, area under the receiver operating curve; LIF, liver inflammation and fibrosis score; Se., sensitivity; Sp., specificity.

a In the steatosis, activity and fibrosis (SAF) score, biopsies are reported for steatosis (0‐3), activity (0‐4; sum of ballooning (0‐2) and lobulitis (0‐2)) and fibrosis (0‐2). The fatty liver inhibition of progression (FLIP) algorithms categorise patients as NASH if all of steatosis, ballooning and lobulitis are graded as 1 or higher and as steatosis if this criterion is not met. The overall disease severity is also classified as mild (fibrosis <2 and activity <2) or significant (fibrosis or activity ≥2).

#### 3.2.2 Transient elastography

There was a significant association between LS and histological fibrosis stage (*r*_s_=.56; *P*=.0003). Patients with cirrhosis had higher median LS compared to patients without cirrhosis (27.0 kPa vs 7.0 kPa; *P*=.002; Figure S3) and the AUROC of TE for the diagnosis of cirrhosis was 0.93 (95% CI: 0.85‐1.00; *P*=.005). A LS cut‐off of 14.7 kPa had a sensitivity of 100% (95% CI: 40%‐100%) and specificity of 91% (95%CI: 76%‐98%, Table S4).

The diagnostic accuracy of the two techniques for the diagnosis of significant (F2‐4) and bridging fibrosis (F3‐4) and cirrhosis (F4) are summarised in Table S4.

### 3.3 Assessment of disease activity; ballooning and lobular inflammation

There was a significant association between histological ballooning grade and the LIF score (*r*_s_=.59; *P*<.0001). The median LIF scores for patients with ballooning grades 0, 1 and 2 were 1.2, 2.7 and 3.5, respectively, with significant differences between all the groups (Figure 3). The area under the receiver operating characteristic curve (AUROC) for the diagnosis of no ballooning vs ballooning grades 1 and 2 was 0.83 (95% CI: 0.72‐0.93; *P*<.0001). A LIF cut‐off of 1.2 had a sensitivity of 91% (95% CI: 82%‐98%) and specificity of 53% (95% CI: 28%‐77%) for the diagnosis of ballooning grade >0 (Table 2).

The median LIF scores of patients with no lobular inflammation and lobular inflammation grade >0 were 1.5 and 2.7 respectively (*P*=.024, Figure 3). There was an association between LIF and overall activity (sum of ballooning + lobular inflammation grades; *r*_s_ .58; *P*<.0001). The median LIF scores for patients with activity scores 0‐1 and 2‐4 were 1.3 and 3.0 respectively (*P*<.0001; Figure 3). The AUROC for the diagnosis of patients with activity ≥2 was 0.83 (95% CI: 0.73‐0.93; *P*<.0001). A LIF cut‐off of 1.6 had a sensitivity of 90% (95% CI: 77%‐97%) and specificity of 61% (95% CI: 39%‐80%) for the diagnosis of an activity grade ≥2 (Table 2).

**Figure 4:**
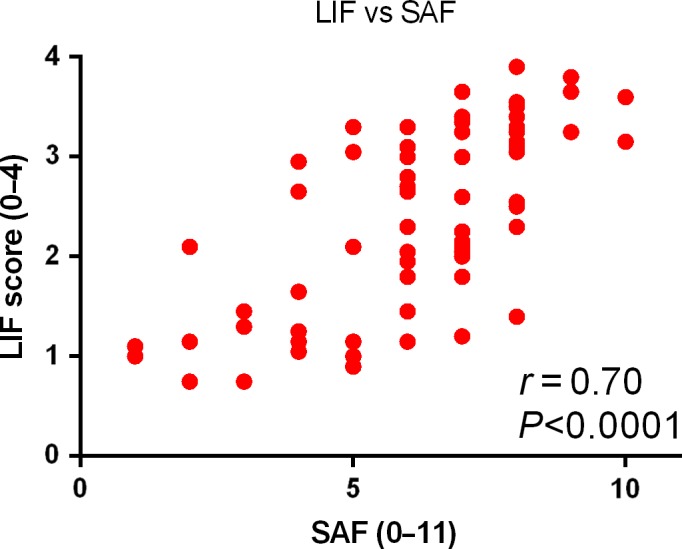
Liver inflammation and fibrosis score for the assessment of the total steatosis, activity and fibrosis score. There was a strong association between the liver inflammation and fibrosis score (LIF) and the overall histological severity scored by the steatosis, activity and fibrosis score (SAF;* r*_s_=.70; *P*<.0001) [Colour figure can be viewed at wileyonlinelibrary.com]

Overall, there was a strong association between the total SAF score and LIF score (*r*_s_=.70, *P*<.0001; Figure 4).

### 3.4 Steatosis vs NASH

**Figure 5:**
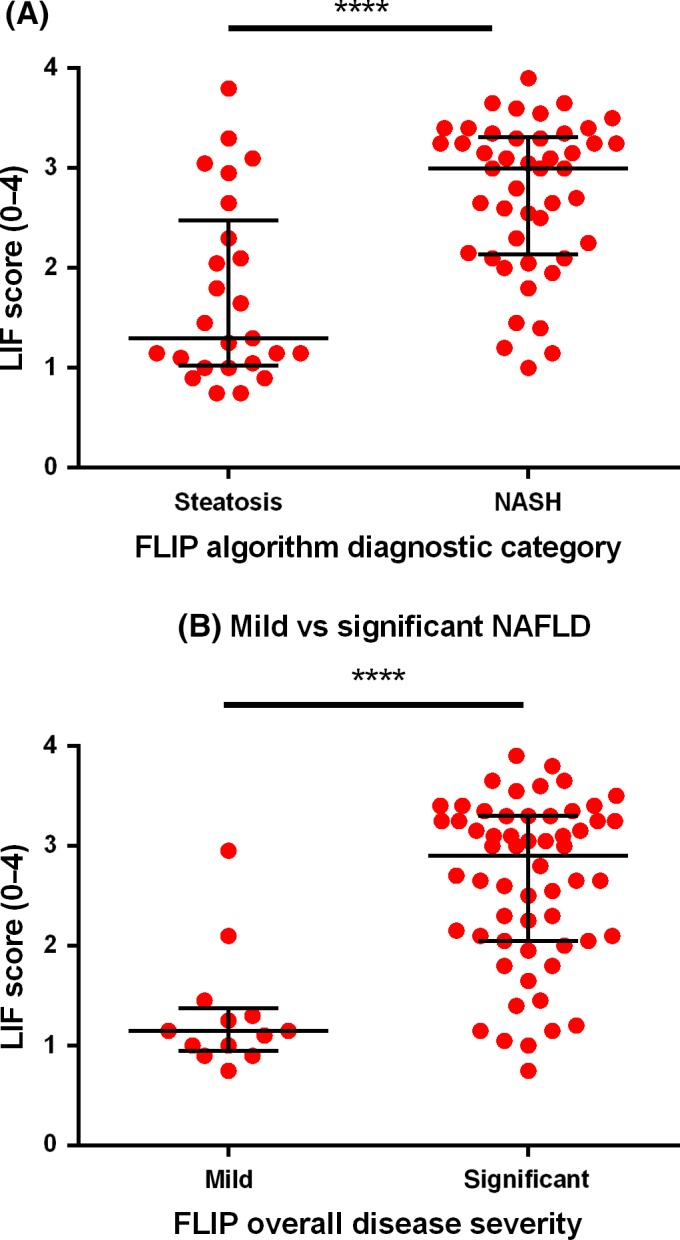
Liver inflammation and fibrosis score for NAFLD classification according to the Fatty Liver Inhibition of Progression consortium algorithms. Liver inflammation and fibrosis scores (LIF) are plotted against the diagnostic categories of the Fatty Liver Inhibition of Progression (FLIP) algorithm. (A), Patients with steatosis had lower median LIF scores compared to patients with NASH (1.3 vs 3.0; *P*<.0001) and (B), patients with mild disease had lower median LIF scores compared to patients with severe disease (2.2 vs 3.3; *P*<.0001). Lines and error bars indicate the median and interquartile range on both graphs [Colour figure can be viewed at wileyonlinelibrary.com]

Patients categorised using the FLIP algorithm into steatosis and NASH had a median LIF of 1.3 and 3.0 respectively (*P*<.0001, Figure 5). The AUROC for the diagnosis of steatosis vs NASH was 0.80 (95% CI: 0.69‐0.92; *P*<.0001). A LIF cut‐off of 1.4 had a sensitivity of 91% (95% CI: 79%‐98%) and specificity of 52% (95% CI: 31%‐72%; Table 2).

### 3.5 Overall disease severity (mild vs significant)

Stratified according to the FLIP consortium algorithm, patients with mild and significant disease had a median LIF 1.2 and 2.9 respectively (*P*<.0001; Figure 5). The AUROC for the diagnosis of mild vs significant NAFLD was 0.89 (95% CI: 0.80%‐0.98%; *P*<.0001) and a LIF cut‐off of 1.4 had a sensitivity of 90% (95% CI: 79%‐96%) and specificity of 77% (95% CI: 46%‐95%; Table 2).

## 4 DISCUSSION

This prospective pilot study has shown that multiparametric MRI can be used to assess the overall disease severity in patients with NAFLD, with an AUROC of 0.89 for the detection of significant disease as defined by the FLIP consortium algorithm. Furthermore, this technique had high diagnostic accuracy for the diagnosis of NASH (AUROC of 0.80) and hepatocyte ballooning (AUROC 0.83), a lesion that is considered central to the diagnosis of NASH.^23^ The accuracy for the diagnosis of cirrhosis was also good with an AUROC of 0.85. Overall, the results suggest that multiparametric MR is sensitive and accurate in quantifying both the inflammatory (NASH/ballooning) and fibrotic components of NAFLD with an excellent diagnostic accuracy for the assessment of overall severity.

Several aspects of this technique would make it attractive as a surrogate end point in clinical trials. The high diagnostic accuracy for both the inflammatory and fibrotic components of NAFLD, combined with the low reporting variability, could mean that small early changes in response to therapy may be more readily detected. This would be particularly important in early trials evaluating anti‐inflammatory and antifibrotic therapies for NAFLD. Recruitment and retention to clinical trials in NAFLD, which rely on liver biopsy to assess end points, have been problematic with a recent study reporting a 25% dropout rate.^24^ Implementation of robust non‐invasive surrogate end points may therefore lead to significant improvements in patient retention and the rapid evaluation of novel therapeutics. MR could also be used in clinical practice for the risk stratification of patients. Potentially, a LIF cut‐off of 1.4 could be used to identify patients with significant NAFLD/NASH who may need follow‐up in specialist clinics or prioritisation for treatment and lifestyle interventions. As multiparametric MRI is completely non‐invasive, it is ideally suited for monitoring disease over time.

Multiparametric MR had a significantly higher success rate (95%) compared to TE (59%, *P*<.0001), while there was a considerable overlap in the 95% confidence intervals for the diagnostic accuracy in the detection of fibrosis, indicating no significant differences between the two techniques (Table S4). TE is solely used for the assessment of fibrosis, so its diagnostic accuracy in the assessment of activity, NASH and overall disease severity was not examined in this study. An advantage of MR technology is that this can detect the early hallmark features of NASH (inflammation and ballooning) before fibrosis is established.

Traditionally MR imaging is used for the assessment of focal liver lesions such as tumours. However, T_1_ mapping techniques are now emerging for the assessment of diffuse liver disease. Studies of T_1_ mapping in animals and humans have shown promising results in the assessment of fibrosis and cirrhosis,^25^, ^26^ and in NAFLD classification.^27^ However, previous studies have largely relied on the use of injectable agents in order to achieve meaningful levels of diagnostic accuracy. The study presented here, is the first to utilise a multiparametric MR approach, where good diagnostic accuracy can be achieved without the need for intravenous agents. We believe that this is possible because our technique removes the confounding effect of iron on T_1_ measures.

Multi‐parametric MR liver assessment can be carried out quickly and would only add 2‐3 minutes to the duration of a standard clinical liver MR examination, something that would incur only minimal costs. A session dedicated solely to run the multiparametric MR protocol would take 10‐15 minutes, allowing for time to get the patient in and out of the scanner. The costs of a dedicated multiparametric MR examination are comparable to the cost of other patented serum and imaging biomarkers. A cost‐benefit analysis was beyond the scope of this work and should be addressed separately in dedicated studies.

The prospective design of this study, where the majority of the MR data were acquired and analysed before liver biopsy is a major strength of this study. The associations between histological variables and the non‐invasive tests evaluated here were moderate, but some aspects of this study may have biased these results. Firstly, this study can only be considered as a pilot study because of the heterogeneity of the included cohorts and the small number of patients. Furthermore, the use of histology as the reference standard is always problematic in studies of diagnostic accuracy of non‐invasive tests because of sampling and observer‐dependent variability of biopsy. This may be a particular issue where there are intermediate quantities of fibrosis and fat. We have previously found good agreement between assessors in our centre for the assessment of fibrosis (weighted kappa 0.51) and steatosis (weighted kappa 0.72).^18^ In addition, for the histological assessment of fibrosis, a minimum biopsy length of 25 mm or at least 11 portal tracts are needed for reliable scoring.^28^ However, a biopsy with at least six portal tracts is generally considered adequate for routine diagnosis.^29^ As our aim was to conduct a comparison with all the histological aspects of NAFLD, we have not excluded any biopsies based on quality criteria as the pathologists could assess all the histological parameters of interest in our study.

In conclusion, this study shows that multiparametric MR is a promising tool for the evaluation of patients with NAFLD. MR gave reliable data more frequently compared to TE, with no differences in the diagnostic accuracy for significant fibrosis or cirrhosis. Furthermore, multiparametric MR had good accuracy for the diagnosis of NASH and ballooning. Therefore, the ability to assess both the necro‐inflammatory and fibrotic components of NASH in a single test is a particular strength of the MR technique that allows accurate evaluation of the overall disease severity. Further refinement and the technical development of non‐invasive biomarkers that enable separate quantification of the inflammatory and fibrotic components of NAFLD is likely to revolutionise this field. Long‐term follow‐up of patients with NAFLD will be required to determine the prognostic capabilities of multiparametric MR, and this should be the focus of future studies.

## CONFLICT OF INTEREST

MP, RB, EMT, CK, MDR, SN and EB are shareholders Perspectum Diagnostics, a university of Oxford spin out company. RB and CK are employed by Perspectum Diagnostics, and RB, MDR and SN are on the board of directors of Perspectum Diagnostics.

## Supporting information

Additional Supporting Information may be found at onlinelibrary.wiley.com/doi/10.1111/liv.13284/suppinfo
