# Supplementary material for: Multiparametric magnetic resonance imaging for the assessment of non‐alcoholic fatty liver disease severity
Source: Liver Int. 2017 May 30;37(7):1065–73. doi: 10.1111/liv.13284 (PMC5518289; doi:10.1111/liv.13284)
Supplement: Supplementary file 1 [file LIV-37-1065-s001.docx]

**Supplement to:** Multi-parametric magnetic resonance imaging for the assessment of non-alcoholic steatohepatitis disease severity

**Authors:** Michael Pavlides, Rajarshi Banerjee, Elizabeth M Tunnicliffe, Catherine Kelly, Jane Collier, Lai Mun Wang, Kenneth A Fleming, Jeremy F Cobbold, Matthew D Robson, Stefan Neubauer, Eleanor Barnes

**Table of contents**

Supplementary methods…………………………………………………………….2

Supplementary tables………………………………………………………………..3

Supplementary figures ………………………………………………………………7

Supplementary references…………………………………………………………..8

**Supplementary Patient and Methods**

*Study design and patient population*

The study included 3 groups of patients.

1. Patients with suspected NAFLD were patients who were planned to have liver biopsy for evaluation of disease stage and diagnosis of NASH. These patients had MRI scan prior to liver biopsy.

2. Patients with known NAFLD. These were patients who had liver biopsy showing NAFLD. These patients had the study MR scan after the biopsy.

3. Bariatric patients were patients who were planned to have bariatric surgery. These patients have a liver biopsy taken at the time of surgery as part of their routine care. They had their study MR scan prior to surgery and liver biopsy.

A subset of the cohort included in this study (17 out of 71 patients) was also included in a study examining the use of multi-parametric MR for the assessment of liver disease in patients with unselected disease aetiologies (1).

*MR Image acquisition*

The imaging protocol included acquisition of localiser images which were used to identify the mid-equatorial transverse liver plane, where T_1_ and T_2_* maps were subsequently acquired. A subject dependent frequency adjustment in end-expiration was performed before the T_1_ and T_2_* acquisitions, which were also acquired in end expiration. Subjects had to hold their breath for 10-12 seconds for each imaging acquisition, and the scanning protocol could be completed in 5 breath-holds (one for localisers, two for T_1_ mapping and two for T_2_* mapping). Image acquisition was ECG gated, with images acquired 200ms after the R-wave. No intravenous contrast injection was used in this protocol.

T_1_ mapping

The shortened Modified Look Locker Inversion recovery (shMOLLI) sequence (2) was used for T_1_ mapping. The ShMOLLI sequence samples the T_1_ recovery curve using single-shot steady state free precession (SSFP) acquisitions. The following parameters were used:

TR 2.14ms, TE 1.07ms, flip angle of 35°, field-of-view optimised per patient, acquisition matrix 192 x 134 to160, depending on patient, GRAPPA acceleration of 2, 24 reference lines, typical interpolated voxel size 0.9 x 0.9 x 8mm. The total time for each SSFP acquisition was between 169 and 197ms, depending on the number of phase encoding steps.

T_2_* mapping

A multi-gradient-echo acquisition with radiofrequency spoiling was used to calculate T_2_*. The same field-of-view as in the T_1_ mapping sequence was used, with a matrix size of 192x128-160, depending on patient, slice thickness of 3mm and 2x GRAPPA acceleration. The image was acquired in nine segments with a TR of 26.5ms and flip angle of 20°. Echo times were selected as far as possible such that the signals from fat and water were in phase (TE = 2.46, 7.38, 12.30, 17.22 and 22.14ms). Fat-saturation and a double-inversion-recovery black blood preparation were used.

*Inter-observer agreement in reporting of MR scans*

A second blinded operator (CK) analysed the first 33 consecutive scans and Bland Altman analysis was performed to assess inter-observer agreement.

**Supplementary Results**

*Inter-observer agreement in reporting of MR scans*

The Bland Altman plot for the agreement between two observers in the reporting of the LIF score is shown in Figure S1. The mean difference between the two measurements was not significantly different from 0 (p=0.31), indicating no bias between the results from the two observers.

**Supplementary Tables**

| **Table S1**: Baseline characteristics for patients with suspected NAFLD (n=48) | |
| --- | --- |
| Age (years; mean ± SD) | 54.4±12.2 |
| Male (n, %) | 35 (70) |
| BMI (kg/m^2^; median; IQR) | 30.4 (27.6 – 33.9) |
| Type 2 Diabetes Mellitus (n, %) | 14 (29) |
| **Liver function tests; median (IQR)** |  |
| Bilirubin (µmol/l) | 12 (7 – 16) |
| ALT (IU/l) | 61 (40 -91) |
| ALP (IU/l) | 181 (148 – 236) |
| Albumin (g/l) | 45 (44 – 47) |
| GGT (IU/l) | 75 (36 – 126) |
| AST (IU/l) | 46 (35 – 63) |
| **Haematological tests; median (IQR)** |  |
| Platelet count (x10^9^/l) | 183 (139 – 232) |
| Prothrombin time (s) | 13.6 (13.1 – 14.4) |
| **Metabolic profile; median (IQR)** |  |
| Glucose (mmol/l) | 5.2 (4.9 – 6.4) |
| Cholesterol (mmol/l) | 4.9 (4.1 – 5.7) |
| HDL (mmol/l) | 1.1 (1.0 – 1.4) |
| LDL (mmol/l) | 2,8 (2.2 – 3.5) |
| Triglycerides (mmol/l) | 1.4 (1.1 – 3.4) |
| **Histology (n, %)** |  |
| **Fibrosis** |  |
| 0 | 5 (10) |
| 1 | 17 (35) |
| 2 | 10 (21) |
| 3 | 8 (17) |
| 4 | 8 (17) |
| **Ballooning** |  |
| 0 | 12 (25) |
| 1 | 28 (58) |
| 2 | 8 (17) |
| **Lobular inflammation** |  |
| 0 | 8 (17) |
| 1 | 40 (83) |
| **Steatosis** |  |
| 0 | 2 (4)* |
| 1 | 4 (8) |
| 2 | 11 (23) |
| 3 | 31 (65) |
| **FLIP algorithm classification (n; %)** |  |
| Steatosis | 17 (35) |
| NASH | 31 (65) |
| Mild disease | 11 (23) |
| Significant disease | 37 (77) |
| **Non-invasive scores; median (IQR)** |  |
| Iron corrected T_1_ (cT_1_; ms) | 936 (822 – 1054) |
| Liver inflammation and fibrosis (LIF) score (0-4) | 2.7 (1.3 – 3.3) |
| Liver stiffness (kPa; n=26) | 7.5 (5.1 – 14.3) |
| *Two patients with suspected NAFLD were found to have no steatosis on liver biopsy but still included as the absence of NAFLD could not have been predicted without liver biopsy, and the patients were recruited prior to biopsy.  Abbreviations: **FLIP**: Fatty Liver Inhibition of Progression consortium, | |
| **Table S2**: Baseline characteristics for patients with known NAFLD (n=11) | |
| Age (years; mean ± SD) | 53.1±10.3 |
| Male (n, %) | 7 (64) |
| BMI (kg/m^2^; median; IQR) | 34.6 (30.1 – 36.7) |
| Type 2 Diabetes Mellitus (n, %) | 7 (64) |
| **Liver function tests; median (IQR)** |  |
| Bilirubin (µmol/l) | 10 (8 – 22) |
| ALT (IU/l) | 60 (31 – 77) |
| ALP (IU/l) | 172 (112 – 248) |
| Albumin (g/l) | 44 (40 – 46) |
| GGT (IU/l) | 89 (44 – 122) |
| AST (IU/l) | 43 (31 – 55) |
| **Haematological tests; median (IQR)** |  |
| Platelet count (x10^9^/l) | 242 (204 – 296) |
| Prothrombin time (s) | 13.3 (12.3 – 14.6) |
| **Metabolic profile; median (IQR)** |  |
| Glucose (mmol/l) | 6.3 (5.1 – 7.5) |
| Cholesterol (mmol/l) | 4.1 (3.5 – 4.9) |
| HDL (mmol/l) | 1.1 (0.9 – 1.4) |
| LDL (mmol/l) | 2.1 (1.7 – 3.1) |
| Triglycerides (mmol/l) | 1.9 (1.5 – 2.1) |
| **Histology (n, %)** |  |
| **Fibrosis** |  |
| 0 | 0 |
| 1 | 1 (10) |
| 2 | 4 (36) |
| 3 | 4 (36) |
| 4 | 2 (18) |
| **Ballooning** |  |
| 0 | 0 |
| 1 | 11 (100) |
| 2 | 0 |
| **Lobular inflammation** |  |
| 0 | 0 |
| 1 | 10 (90) |
| 2 | 1 (10) |
| **Steatosis** |  |
| 0 | 0 |
| 1 | 0 |
| 2 | 2 (18) |
| 3 | 9 (82) |
| **FLIP algorithm classification (n; %)** |  |
| Steatosis | 0 |
| NASH | 11 (100) |
| Mild disease | 0 |
| Significant disease | 11 (100) |
| **Non-invasive scores; median (IQR)** |  |
| Iron corrected T_1_ (cT_1_; ms) | 917 (858 – 1015) |
| Liver inflammation and fibrosis (LIF) score (0-4) | 2.6 (1.8 – 3.2) |
| Liver stiffness (kPa; n=9) | 7.8 (5.5 – 14.0) |
| Abbreviations: **FLIP**: Fatty Liver Inhibition of Progression consortium | |

| **Table S3**: Baseline characteristics for patients undergoing bariatric surgery (n=12) | |
| --- | --- |
| Age (years; mean ± SD) | 49.8 ± 9.6 |
| Male (n, %) | 1 (8) |
| BMI (kg/m^2^; median; IQR) | 47.6 (41.1 – 52.1) |
| Type 2 Diabetes Mellitus (n, %) | 4 (33) |
| **Liver function tests; median (IQR)** |  |
| Bilirubin (µmol/l) | 8 (6 – 13) |
| ALT (IU/l) | 23 (18 – 42) |
| ALP (IU/l) | 135 (91 – 156) |
| Albumin (g/l) | 45 (44 – 47) |
| GGT (IU/l) | 33 (17 – 53) |
| AST (IU/l) | 29 (21 – 35) |
| **Haematological tests; median (IQR)** |  |
| Platelet count (x10^9^/l) | 299 (268 – 314) |
| Prothrombin time (s) | 14.3 (13.1 – 14.7) |
| **Metabolic profile; median (IQR)** |  |
| Glucose (mmol/l) | 4.6 (4.4 – 5.0) |
| Cholesterol (mmol/l) | 3.9 (3.4 – 5.4) |
| HDL (mmol/l) | 1.1 (0.9 – 1.2) |
| LDL (mmol/l) | 2.1 (1.4 – 3.4) |
| Triglycerides (mmol/l) | 1.7 (1.2 – 1.8) |
| **Histology (n, %)** |  |
| **Fibrosis** |  |
| 0 | 0 |
| 1 | 2 (17) |
| 2 | 6 (50) |
| 3 | 3 (25) |
| 4 | 1 (8) |
| **Ballooning** |  |
| 0 | 5 (42) |
| 1 | 7 (58) |
| 2 | 0 |
| **Lobular inflammation** |  |
| 0 | 4 (33) |
| 1 | 8 (67) |
| 2 | 0 |
| **Steatosis** |  |
| 0 | 2 (17)* |
| 1 | 4 (33) |
| 2 | 4 (33) |
| 3 | 2 (17) |
| **FLIP algorithm classification (n; %)** |  |
| Steatosis | 8 (67) |
| NASH | 4 (33) |
| Mild disease | 2 (17) |
| Significant disease | 10 (83) |
| **Non-invasive scores; median (IQR)** |  |
| Iron corrected T_1_ (cT_1_; ms) | 886 (816 – 976) |
| Liver inflammation and fibrosis (LIF) score (0-4) | 2.1 (1.2 – 3.0) |
| Liver stiffness (kPa; n=3) | 6.4 (4.8 – 6.6) |
| *Two patients were found to have no steatosis on liver biopsy but still included as the absence of NAFLD could not have been predicted without liver biopsy, and the patients were recruited prior to biopsy.  Abbreviations: **FLIP**: Fatty Liver Inhibition of Progression consortium | |

| **Table S4:** Diagnostic accuracy of multi-parametric magnetic resonance imaging and transient elastography for the assessment of fibrosis in patients with NAFLD | | | | | | | | |
| --- | --- | --- | --- | --- | --- | --- | --- | --- |
|  | **LiverMultiScan (n=71)** | | | | **Transient elastography(n=38)** | | | |
|  | AUROC  (95% CI) | Cut-off  (0.0-4.0) | Se.  (%) | Sp.  (%) | AUROC  (95% CI) | Cut-off  (kPa) | Se.  (%) | Sp.  (%) |
| F0-1 vs F2-4 | 0.78  (0.67 – 0.89) | 1.2 | 91 | 36 | 0.73  (0.58 – 0.90) | 3.8 | 92 | 17 |
| F0-2 vs F3-4 | 0.73  (0.61 – 0.85) | 1.7 | 92 | 40 | 0.83  (0.7 – 0.96) | 6.8 | 93 | 65 |
| F0-3 vs F4 | 0.85  (0.76 – 0.95) | 3.0 | 91 | 73 | 0.93  (0.85 – 1.00) | 14.7 | 100 | 91 |
| Abbreviations. **AUROC:** area under the receiver operating characteristic curve, **95% CI:** 95% confidence interval, **Se:** sensitivity, **Sp:** specificity | | | | | | | | |

**Supplementary Figures**

**Figure S1. Bland Altman plot for inter-observer agreement in MR reporting.**

The mean of the difference between the two observers was -0.106, and it was not significantly different from 0 (p=0.31), indicating no inter-observer bias. The 95% levels of agreement were between 1.061 and -1.258.

26

12

11

M probe attempted (n=23)

M probe not attempted (n=41)

**M probe success rate 12/23 (52%)**

XL probe attempted (n=52)

**XL probe success rate 26/52 (50%)**

Valid measurements in 38 / 64 patients

**Overall success rate (58%)**

Patients in whom TE was attempted (n=64)

**Figure S2: Transient elastography success rates.**

The M probe was used first in 23 patients. Of these, 12 had a successful liver stiffness measurement (M probe success rate 52%), and 11 went on to have an examination with the XL probe. An additional 41 patients had an examination only with the XL probe. XL examination was successful in 26 patients (XL probe success rate 50%). Overall, a successful liver stiffness measurement was obtained in 38 patients

**Figure S3: Transient elastography for the assessment of fibrosis in NAFLD.**

There was a significant correlation between liver stiffness and fibrosis (r_s_=0.56; p=0.0003). The median liver stiffness for patients with cirrhosis (27.0kPa) was higher than patients without cirrhosis (7.0kPa, p=0.005)

Lines and error bars indicate the median and interquartile range.

**Supplementary References**

1. BANERJEE R, PAVLIDES M, TUNNICLIFFE E M, et al. Multiparametric magnetic resonance for the non-invasive diagnosis of liver disease. J Hepatol 2014; 60(1): 69-77.

2. PIECHNIK S K, FERREIRA V M, DALL'ARMELLINA E, et al. Shortened Modified Look-Locker Inversion recovery (ShMOLLI) for clinical myocardial T1-mapping at 1.5 and 3 T within a 9 heartbeat breathhold. J Cardiovasc Magn Reson 2010; 12: 69.
